# Risk factors for mortality among patients diagnosed with multi-drug resistant tuberculosis in Uganda- a case-control study

**DOI:** 10.1186/s12879-021-05967-2

**Published:** 2021-03-22

**Authors:** Enock Kizito, Joseph Musaazi, Kenneth Mutesasira, Fred Twinomugisha, Helen Namwanje, Timothy Kiyemba, Debora B. Freitas Lopez, Nicholas Sebuliba Nicholas, Abel Nkolo, Estella Birabwa, Seyoum Dejene, Stella Zawedde-Muyanja

**Affiliations:** 1 USAID/Defeat TB Project, University Research Co. LLC, Kampala, Uganda; 2 The Infectious Diseases Institute, College of Health Sciences, Makerere University, P.O. Box 22418, Kampala, Uganda; 3 University Research Co. LLC, Chevy Chase, Maryland USA; 4 USAID, Kampala, Uganda

**Keywords:** Tuberculosis, Multidrug-resistance, Mortality, Uganda

## Abstract

**Background:**

The World Health Organization (WHO) End TB strategy aims to reduce mortality due to tuberculosis (TB) to less than 5% by 2035. However, mortality due to multidrug-resistant tuberculosis (MDR-TB) remains particularly high. Globally, almost 20% of patients started on MDR-TB treatment die during the course of treatment every year. We set out to examine the risk factors for mortality among a cohort of patients diagnosed with MDR-TB in Uganda.

**Methods:**

We conducted a case-control study nested within the national MDR-TB cohort. We defined cases as patients who died from any cause during the course of MDR-TB treatment. We selected two controls for each case from patients alive and on MDR-TB treatment at the time that the death occurred (incidence-density sampling). We matched the cases and controls on health facility at which they were receiving care. We performed conditional logistic regression to identify the risk factors for mortality.

**Results:**

Data from 198 patients (66 cases and 132 controls) started on MDR-TB treatment from January 1 to December 31, 2016, was analyzed for this study. Cases were similar to controls in age/sex distribution, occupation and history of TB treatment. However, cases were more likely to be HIV infected while controls were more likely to have attained secondary level education. On multivariate regression analysis, co-infection with HIV (aOR 1.9, 95% CI [1.1–4.92] *p* = 0.05); non-adherence to MDR-TB treatment (aOR 1.92, 95% CI [1.02–4.83] *p* = 0.04); age over 50 years (aOR 3.04, 95% CI [1.13–8.20] *p* = 0.03); and having no education (aOR 3.61, 95% CI [1.1–10.4] *p* = 0.03) were associated with MDR-TB mortality.

**Conclusion:**

To mitigate MDR-TB mortality, attention must be paid to provision of social support particularly for older persons on MDR-TB treatment. In addition, interventions that support treatment adherence and promote early detection and management of TB among HIV infected persons should also be emphasized.

## Background

Multi-drug resistant tuberculosis (MDR-TB), defined as tuberculosis that is resistant to at both rifampicin and isoniazid, two first line anti-tuberculous drugs [1, 2], is an ongoing global public health challenge. In 2019, the World Health Organization (WHO) estimated that only about 35% of the estimated 500,000 incident cases of MDR-TB were started on an appropriate MDR-TB treatment regimen [3] Among cohorts of patients on treatment, treatment outcomes have consistently been suboptimal. In 2019, only 57% of patients started on MDR-TB treatment 2 years earlier (2017) successfully completed treatment [3]. Mortality while on MDR-TB treatment accounted for 15% of all patients who did not complete treatment, the highest case fatality ratio being in the African region at 18% [3].

Uganda is among the 30 high TB-HIV burden countries in the world. The country has an estimated TB prevalence of 253/100,000 population and about 2000 incident MDR-TB cases annually [4]. Due to limited access to rapid molecular diagnostic tests, MDR-TB case notification continues to be suboptimal and in 2019, the country notified only 541 MDR-TB cases (25% of the estimated incident cases) [5]. HIV co-infection among MDR-TB patients remains high (30% MDR-TB/HIV co-infection in the 2019 cohort) and like the rest of the world, treatment outcomes are suboptimal. Only two thirds of those started on MDR-TB treatment in 2017 successfully completed treatment with 20% of all patients dying during the course of therapy [6].

Although suboptimal treatment outcomes among patients started on MDR-TB treatment are a cause of universal concern, studies examining risk factors for these suboptimal outcomes are few and are mostly from clinical research studies in high resource settings [7, 8]. However, we understand that patient characteristics and modalities for provision of care in programmatic settings may be markedly different from that in clinical research settings. We therefore set out to examine the risk factors for mortality among a cohort of patients diagnosed with MDR-TB and treated in a programmatic setting in Uganda.

## Methods

### Study setting

The Uganda national MDR-TB cohort consists of all patients initiated on MDR-TB treatment in the country in a given year. For this cohort, patients diagnosed with rifampicin resistant TB on the Xpert® MTB/RIF assay are referred to any of the tertiary referral hospitals with MDR-TB treatment facilities. On arrival at these hospitals, additional baseline investigations are performed including sputum culture, drug susceptibility testing, chest X-rays, HIV tests, thyroid function tests and blood chemistry tests. Patients are then started on the national standardized MDR-TB treatment regimen while awaiting sputum culture and drug susceptibility testing results in keeping with the Uganda national guidelines for the programmatic management of drug resistant TB (PMDT) [2]. On receiving drug susceptibility test results, patients either continue with their treatment (if found to have resistance to rifampicin +/− isoniazid) or are given individualized regimens if found to be resistant to fluoroquinolones. In 2016, the national standardized treatment regimen for MDR-TB consisted of six to eight months of Kanamycin, Levofloxacin, Cycloserine, Ethionamide, and Pyrazinamide followed by 14 months of Levofloxacin, Cycloserine, Ethionamide, and Pyrazinamide [2]. Patients with resistance to fluoroquinolones or injectable agents were initiated on alternative individualized regimens comprising of bedaquiline for 6 months and other companion drugs depending on their susceptibilty profiles, as guided by the national panel for a total duration of 24 months. Following initiation of treatment, patients are placed on daily directly observed therapy (DOT) at the MDR-TB treatment facility or at a primary healthcare facility - usually closer to the patient’s home - manned with staff trained in MDR-TB care who receive periodic mentorship from the tertiary treatment facility. All patients report back to the tertiary treatment facility once every month. At these monthly follow-up visits, adherence to treatment is assessed and clinical, biochemical, and bacteriological improvement is measured. Adherence is assessed by reviewing  patients'  MDR-TB treatment cards which are filled by the primary care facility staff on each day that DOT is dispensed. Clinical improvement is measured through vital measurement such as weight and blood pressure, while biochemical improvement is measured through blood chemistry tests, e.g. complete blood counts and liver and renal function tests. Finally, bacteriological improvement is measured through sputum smears and cultures. At these visits, patients are also offered social support to cover costs for food and transportation.

### Study participants

**Fig. 1:**
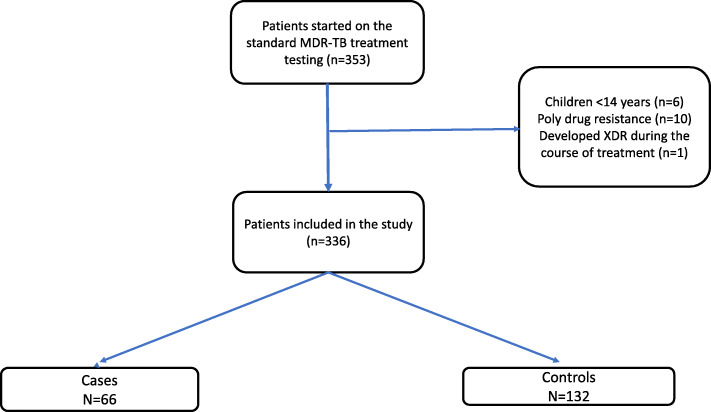
Patient Flow Chart

We conducted a case-control study nested within the 2016 Uganda national MDR-TB cohort. We included only patients aged 15 years and above with confirmed resistance to rifampicin+/− isoniazid on drug susceptibility testing who were initiated on the standard MDR-TB treatment regimen from January 1 to December 31, 2016. We defined cases as patients who died from any cause during the course of TB treatment consistent with Ugandan and international definitions of TB death [2, 9]. We selected two controls for each case from patients alive and on MDR-TB treatment at the time that the death occurred (incidence-density sampling) and matched the cases and controls at the health facility at which they received care (Fig. 1).

### Data collection

We used a standardized case report form to abstract data from patient medical charts. We collected information about sociodemographic characteristics like sex, age, education, marital status, and occupation; clinical characteristics like HIV co-infection, TB treatment history, co-morbidity, CD4 cell counts at MDR-TB diagnosis and behavioral factors like adherence to MDR-TB treatment and use of recreational drugs/alcohol. We checked the data for completeness, entered it into an electronic database system (DHIS II) and then exported it to Microsoft Excel, which was then imported into STATA version 14.0 for analysis.

### Statistical analysis

We described patient characteristics using counts and percentages, and compared the differences in these characteristics between cases and controls using the McNemar chi-square test. We fitted conditional logistic regression to assess risk factors for mortality among patients initiated on MDR-TB treatment. Factors which had *p*-value < 0.2 on bi-variate analysis were entered into a multivariate conditional logistic regression model. Variables with p-value ≤0.05 on multivariate regression were considered as statistically significant risk factors for MDR-TB mortality.

## Results

In 2016, 353 people were started on the standard MDR-TB treatment regimen. By December 2018, 66 patients had died while on MDR-TB treatment, 54 were LFU and 229 were successfully completed treatment and 4 were not evaluated. We selected for this study, all 66 patients who died during TB treatment along with 132 controls. Cases were similar to controls in age/sex distribution, occupation and history of TB treatment. However, cases were more likely to be HIV infected while controls were more likely to have a secondary level education. (Table 1).

**Table 1:** Characteristics of participants enrolled in the study

Characteristics	Total	Cases (***n*** = 66) n (%)	Controls (***n*** = 132) n (%)	***P***-Value^**§**^
**Sex**				0.98
Male	120 (60.6)	40 (60.6)	80 (60.6)	
Female	78 (39.4)	26 (39.4)	52 (39.4)	
**Age**				0.09
0–18	9 (4.6)	1 (1.52)	8 (6.1)	
19–34	91 (46.0)	32 (48.5)	59 (44.7)	
35–49	74 (37.4)	21 (31.8)	53 (40.2)	
50+	24 (12.0)	12 (18.2)	12 (9.1)	
**Education**				0.02
None	56 (28.3)	24 (36.4)	32 (24.2)	
Primary	78 (39.4)	28 (42.4)	50 (37.9)	
Secondary & above	64 (32.3)	14 (21.2)	50 (37.9)	
**Occupation**				0.51
Unskilled work	166 (83.8)	57 (86.4)	109 (82.6)	
Skilled work	32 (16.2)	9 (13.6)	23 (17.4)	
**Previous history of TB¶**				0.80
Yes	108 (55.1)	35 (53.8)	73 (55.7)	
No	88 (44.9)	30 (46.2)	58 (44.3)	
**HIV Status¶**				0.04
Positive	118 (59.6)	45 (70.3)	73 (55.3)	
Negative	78 (40.4)	19 (29.7)	59 (44.7)	
**CD4 Count¶**				0.48
< 200	28 (71.8)	11 (78.6)	17 (68.0)	
≥ 200	11 (28.2)	3 (21.4)	8 (32.0)	
**Adherence to ART**				0.99
Good	195 (98.5)	65 (98.5)	130 (98.5)	
Poor	3 (1.5)	1 (1.5)	2 (1.5)	
**Documented medical complication**^**b**^				
Yes	102 (51.5)	39 (59.1)	63 (47.7)	0.03
No	96 (48.5)	27 (40.9)	69 (52.3)	
**Missed DR-TB doses**^**a**^				
Yes	126 (63.6)	38 (57.6)	88 (66.7)	0.01
No	72 (36.4)	28 (42.4)	44 (33.3)	
**Type of health facility for follow-up care**				
MDR-TB treatment site	111 (56.1)	27 (40.9)	84 (63.6)	
Primary care health facility	87 (43.9)	39 (59.1)	48 (36.4)	0.71

¶Missing data; Previous history of TB (cases = 1, control = 1), HIV status (cases = 2, control = 0), baseline CD4 count (cases = 31, controls = 38) ^§^McNemar Chi-square *P*-value comparing cases and controls

^a^missed DR-TB doses were assessed by checking the client’s DR-TB treatment card held filled at the DOT facility.

^b^The commonest medical complications were anaemia, malnutrition and respiratory distress

On bivariate analysis, risk factors for mortality while on MDR-TB treatment were; patients' education level OR 3.70, 95% CI [1.5–8.0] *p* = 0.02; age >50 years OR 2.51, 95% CI [0.98–6.42] *p* = 0.06; HIV co-infection OR 1.83, 95% CI [0.86–2.70] *p* = 0.07; having missed doses 1.71, 95% CI [0.6–3.40] *p* = 0.22 and having a documented medical complication OR 1.82, 95% CI [0.97–3.40] *p* = 0.05 (Table 2).

**Table 2:** Conditional (fixed effects) logistic regression model of the risk factors associated with the mortality among patients of MDR-TB

Characteristics	Un adjusted OR (95% CI)	***P***-Value	Adjusted OR (95% CI) ƚ	***P***-Value
**Age**				
0–18	[1]		[1]	
19–34	0.22 [0.02–1.82]	0.16	0.18 [0.02–1.70]	0.14
35–49	0.29 [0.04–2.46]	0.26	0.28 [0.03–2.61]	0.27
50+	**2.51 [0.98–6.42]**	**0.06**	**3.04 [1.13–8.20]**	**0.03**
**Education**				
None	**3.70 [1.5–8.0]**	**0.02**	**3.61 [1.1–10.4]**	**0.03**
Primary	1.30 [0.58–2.6]	0.19	2.01 [0.6–4.30]	0.14
Secondary & above	[1]		[1]	
**HIV status**				
Negative	[1]		[1]	
Positive	**1.83 [0.86–2.70]**	**0.07**	**1.9 [1.1–4.92]**	**0.05**
**Documented medical complication**				
No	[1]			
Yes	1.82 [0.97–3.40]	0.06	2.03 [0.67–2.95]	0.09
**Missed DR-TB doses**				
No	[1]		[1]	
Yes	1.71 [0.6–3.40]	0.22	**1.92 [1.02–4.83]**	**0.04**

*OR* Odds Ratio, *CI* Confidence interval. ƚ model fitted on complete records on all variables in the model (total = 190, cases = 64, controls = 126). Data was missing on HIV status on 2 cases, thus their controls were automatically dropped from the model.

On multivariate analysis, risk factors for mortality included not having any education (adjusted odds ratio [aOR] 3.61, 95% CI [1.1–10.4] *p* = 0.03); missing doses (aOR 1.92, 95% CI [1.02–4.83] *p* = 0.04); age above 50 years (aOR 3.04, 95% CI [1.13–8.20] *p* = 0.03) and co-infection with HIV (aOR 1.9, 95% CI [1.1–4.92] *p* = 0.05). (Table 2).

## Discussion

In this study, we sought to determine the risk factors for mortality among patients started on MDR-TB treatment under programmatic conditions in a resource limited setting. We employed a case-control study nested within the 2016 national MDR-TB cohort. We found that being co-infected with HIV and being non-adherent to treatment doubled the risk of death while older age (> 50 years) tripled the risk of mortality from MDR-TB. Having no education was the greatest risk factor for mortality, increasing the risk of death by almost four times.

HIV infection has been previously shown to result in an increase in mortality among persons diagnosed with MDR-TB with this effect increasing with advancing HIV disease [10–12]. In one study from South Africa, patients with CD4 cell counts < 50 cells/mm^3^ had a four-fold increase in mortality from MDR-TB compared to the general population. In our study, although three quarters of all HIV + ve cases and controls presented with advanced HIV disease - defined as CD4 < 200 cells/mm^3^ [13]- the increase in mortality among HIV co-infected patients was lower than has been previously documented [10]. This was probably because all patients in our study were initiated on antiretroviral therapy which has been shown to decrease TB associated mortality [14, 15]. Other measures to decrease mortality among HIV infected MDR-TB patients would include community interventions to promote earlier HIV diagnosis and early return to care for those who interrupt treatment. In addition, efforts to improve the management of patients with advanced HIV disease including screening for TB among these patients should be implemented at health facilities [9].

The standard treatment regimen for MDR-TB used in this study was a minimum of 20 months and consisted of 6 months of injectable medicine [2, 16]. Adherence to this regimen was suboptimal globally with over one third of patients being nonadherent to therapy [17]. In our study, 55% of patients were nonadherent to the TB treatment. Apart from adverse drug reactions associated with this regimen, additional reasons for nonadherence to treatment in our setting may be socioeconomic factors e.g., lack of transport fares to receive health facility DOT and patient exhaustion given the long duration of treatment.

Older age has been associated with increased mortality from TB due to atypical presentations, increasing co-morbidities and more frequent drug related adverse events [18, 19]. In our setting, older age has also been shown to be associated with decreased access to TB care services. The 2015 national TB prevalence survey found that one of the largest prevalence to notification gaps was among persons 50 years and older [4]. Older persons are also less likely to afford daily transport fares for health facility-based DOT making them susceptible to suboptimal adherence to treatment.

**Fig. 2:**
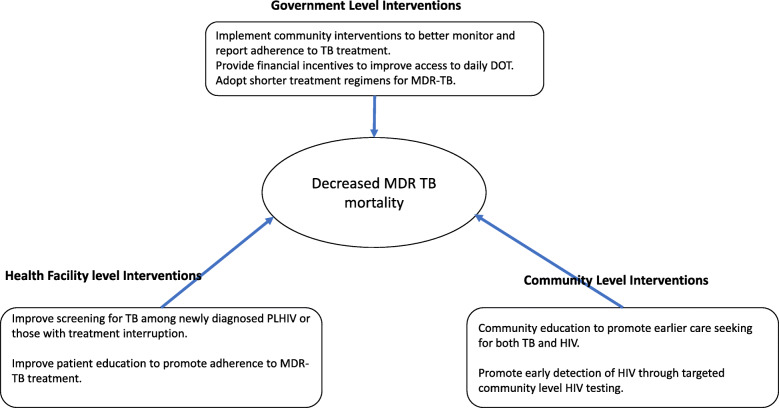
Summary of Proposed Interventions to mitigate MRD-TB mortality

In our study, having no education was the strongest risk factor for mortality during MDR-TB treatment. Compared to patients with a secondary level education, the odds of mortality while on MDR-TB treatment doubled among those with only a primary level education and tripled among those with no education. Globally, persons with more years of schooling are more likely to be employed, have higher incomes and healthier lifestyles [20, 21]. Similarly in our setting, lower education levels are associated with unemployment, poorly paid work, and low social economic status [22]. In our study, 71.4% of patients with no education were unemployed or doing peasantry agriculture versus 37.8% with secondary education. Low social economic status has been associated with an increased likelihood of TB and HIV infection and with poorer outcomes from both diseases [23, 24]. In addition, accessing diagnosis and treatment for MDR-TB has been associated with catastrophic costs to patients and their families [25, 26] which are likely to severely affect patients with low socioeconomic status making it difficult for them to adhere to daily DOT and monthly refill visits. This is seen from the fact that a higher proportion of patients with no education 23/33 (69.7%) missed at least one dose of MDR-TB treatment compared to 19/40 (47.5%) with a secondary education. Financial incentives to ease access to daily DOT among older patients and patients with low education levels as well as community interventions to improve monitoring and reporting of TB treatment adherence may decrease mortality among these patients. The summary of interventions needed to mitigate MDR-TB associated mortality is included in Fig. 2.

Although our study was nationally representative of patients diagnosed with MDR-TB in Uganda and therefore adequately reflects of the risk factors for mortality among these patients, it had several limitations. The use of routinely collected data resulted in missing information in patient files which was minimized by triangulating several data sources. Secondly, some of the variables extracted from the patient charts e.g., alcohol/ recreational drug use were self-reported and prone to information bias as patients would have been reluctant to report undesirable behavior to their healthcare providers. Finally, the study population was chosen from a national cohort in which 15% of patients were lost to follow-up during treatment. It is therefore likely that a proportion of these patients died and that cases were underrepresented in the study which could have resulted in missed associations.

## Conclusion

To improve mitigate MDR-TB mortality, attention must be paid to provision of social support particularly for older persons on MDR TB treatment and persons with low education levels. Interventions that support treatment adherence for all patients diagnosed with MDR-TB should also be  implemented. Finally, early detection of TB among patients with HIV infection should also be emphasized.

## Data Availability

The datasets during and/or analyzed during the current study available from the corresponding author on request.
